# Site-specific and kinetic characterization of enzymatic and nonenzymatic protein acetylation in bacteria

**DOI:** 10.1038/s41598-017-13897-w

**Published:** 2017-11-01

**Authors:** Miao-Miao Wang, Di You, Bang-Ce Ye

**Affiliations:** 1 Collaborative Innovation Center of Yangtze River Delta Region Green Pharmaceuticals, College of Pharmaceutical Sciences, Zhejiang University of Technology, Hangzhou, 310014 Zhejiang, China; 2 Lab of Biosystems and Microanalysis, State Key Laboratory of Bioreactor Engineering, East China University of Science and Technology, Shanghai, 200237 China

## Abstract

Reversible N^ε^-lysine acetylation has emerging as an important metabolic regulatory mechanism in microorganisms. Herein, we systematically investigated the site-specific and kinetic characterization of enzymatic (lysine acetyltransferase) and nonenzymatic acetylation (AcP-dependent or Acyl-CoA-dependent), as well as their different effect on activity of metabolic enzyme (AMP-forming acetyl-CoA synthetase, Acs). It was found that *Bacillus subtilis* acetyl-CoA synthetase (*Bs*AcsA) can be acetylated *in vitro* either catalytically by lysine acetyltransferase *Bs*AcuA and Ac-CoA (at low concentration), or nonenzymatically by Ac-CoA or AcP (at high concentration). Two distinct mechanisms show preference for different lysine acetylation site (enzymatic acetylation for K549 and nonenzymatic acetylation for K524), and reveal different dynamics of relative acetylation changes at these lysine sites. The results demonstrated that lysine residues on the same protein exhibit different acetylation reactivity with acetyl-phosphate and acetyl-CoA, which was determined by surface accessibility, three-dimensional microenvironment, and pKa value of lysine. Acetyl-CoA synthetase is inactivated by AcuA-catalyzed acetylation, but not by nonenzymatic acetylation.

## Introduction

Reversible N^ε^-lysine acetylation, which can change protein conformation, protein charge, protein stability, protein-protein interactions, protein-DNA binding affinity, or protein localization, is an abundant post-translational modification (PTM) in cells of all domains of life. Thousands of acetylated proteins were identified to be involved in diverse cellular processes, such as translation, transcription, and metabolism. Compelling evidence shows that protein lysine acetylation can occur via two distinct mechanisms: enzymatic and nonenzymatic acetylation (chemical acetylation). Enzymatic acetylation relies on a protein acetyltransferase (PAT) to catalyze donation of the acetyl group from acetyl-coenzyme A (Ac-CoA) to the ε-amino group of a lysine residue^1–3^. Recent studies have shown that acetyl phosphate (AcP), the high-energy intermediate of the phosphotransacetylase-acetate kinase pathway, can chemically acetylate lysine residues of protein directly^4,5^. Based on experimental data in *Escherichia coli*, nonenzymatic acetylation with AcP is more global and less specific than enzymatic acetylation, and is thought to predominate in affecting the levels of protein acetylation^4^. Deletion of acetate kinase, which increases intracellular AcP, increase protein lysine acetylation. In contrast, knockout of phosphotransacetylase, which converts Ac-CoA into AcP, decreases protein acetylation. This finding indicates that the intracellular AcP concentration is correlated with protein acetylation levels^4^. Lysine acetylation (or succinylation) has been proposed to also occur by Acyl-CoA-dependent chemical acylation, particularly within the mitochondria, in which the relatively high pH and Ac-CoA (or succinyl-CoA) concentrations would favour transferring acyl group to the deprotonated ε-amino group of lysines. Acyl-CoA-dependent nonenzymatic acylation results in substantial spontaneous mitochondrial protein lysine acetylation or succinylation^6^.

Two distinct mechanisms of lysine acetylation produce the same result, acetylation of ε-amino group of lysine within the target protein. Recently, the structural characterization and physiological function of two acetylation mechanisms was investigated. The previously identified bacterial acetyltransferases only recognize and acetylate the conserved motif (PXXXXGK) which is important for catalytic activity in these AMP-forming acyl-CoA synthetases, and therefore, acetylation inhibits their activity^7^. Mass spectrometric and crystallographic methods revealed AcP-dependent nonenzymatic acetylation to be specific: with specificity determined by the accessibility and three-dimensional microenvironment of the target acetyllysine^5^. Kuhn *et al*. found that an AcP-acetylatable lysine tended to be located near protein surfaces with microenvironment enriched for residues with positive charges, hydroxyls or amides, but often with an adjacent negatively charged residue. More recently, results from studies by Schilling *et al*. demonstrated that individual lysine sites from the same protein not only exhibit different susceptibilities to acetylation, but can also show markedly different acetylation rates in wild-type *E. coli* K-12 during the glucose time course experiments^8^. Nevertheless, the structural characterization and physiological function of two distinct mechanisms of lysine acetylation remain poorly understood yet.

In this work, we systematically investigated the site-specific and kinetic characterization of enzymatic and nonenzymatic acetylation (AcP-dependent or Acyl-CoA-dependent), as well as their different effect on activity of metabolic enzyme (AMP-forming acetyl-CoA synthetase, Acs). It was found that *Bacillus subtilis* acetyl-CoA synthetase (*Bs*AcsA) can be acetylated *in vitro* either catalytically by lysine acetyltransferase *Bs*AcuA and Ac-CoA (at low concentration), or nonenzymatically by Ac-CoA or AcP (at high concentration). Two distinct mechanisms show preference for different lysine acetylation site, and reveal different dynamics of relative acetylation changes at these lysine sites. Acetyl-CoA synthetase is inactivated by AcuA-catalyzed acetylation, but not by nonenzymatic acetylation.

## Results and Discussion

### Acetyl phosphate or acetyl-coenzyme A directly acetylates acetyl-coenzyme A synthetase

**Figure 1:**
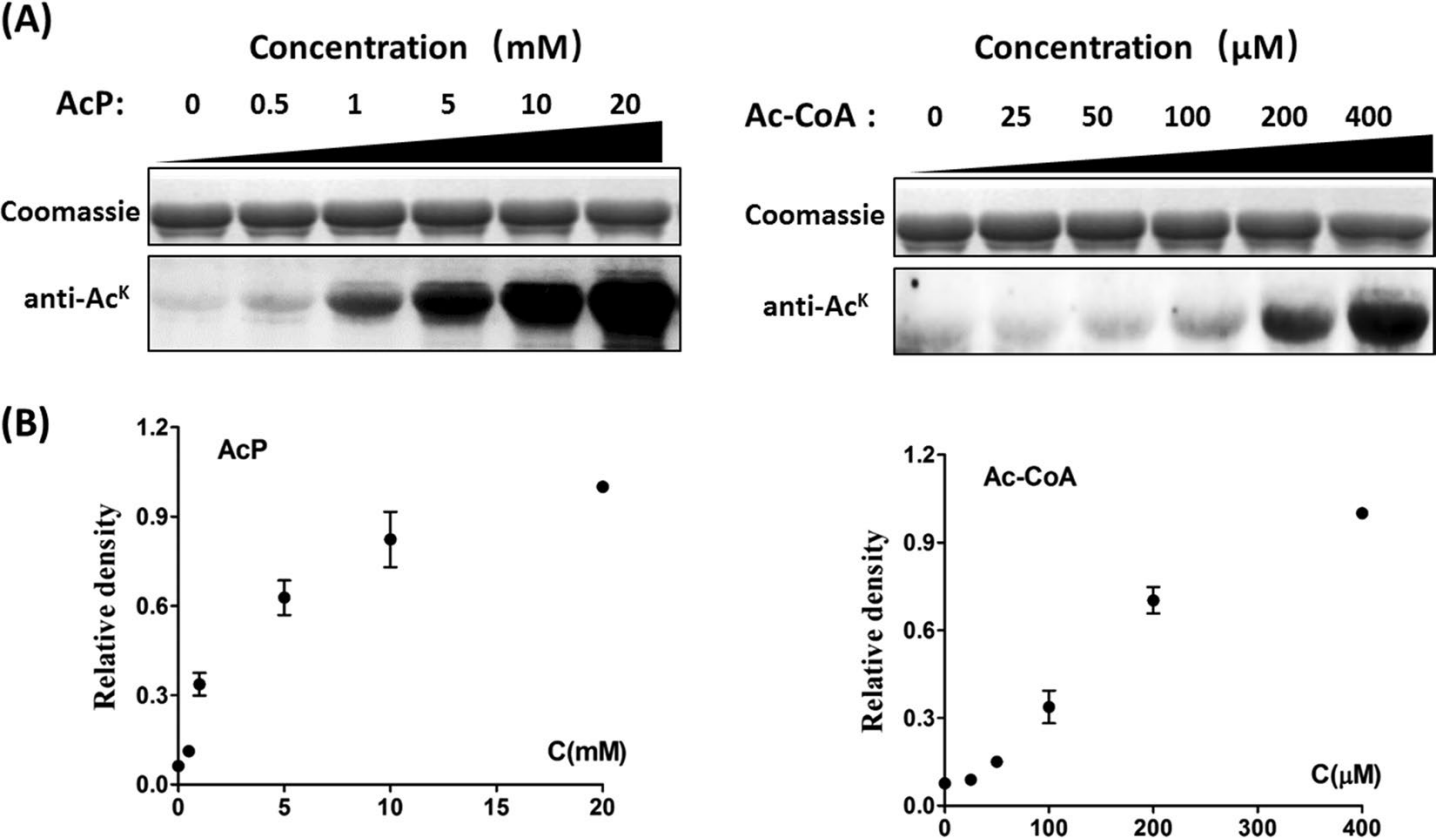
*In vitro* nonenzymatic acetylation of *Bs*AcsA using AcP or Ac-CoA as the acetyl group donor at the different concentrations. (**A**) Coomassie stain and anti-acetyllysine (anti-Ac^K^) Western blot analysis of *Bs*AcsA incubated with AcP (0, 0.5, 1, 5, 10, 20 mM) for 6 h at 37 °C or with Ac-CoA (0, 25, 50, 100, 200, 400 μM) for 6 h at 37 °C. (**B**) Acetylation signals were quantified using Image J software and normalized to the signal at concentration of AcP (20 mM) or Ac-CoA (400 μM).

The best-studied acetylated protein is AMP-forming Ac-CoA synthetase (AcsA), which synthesizes Ac-CoA from acetate and CoA along with the hydrolysis of ATP to AMP. The activity of this enzyme is inhibited by Pat/AcuA-catalyzed acetylation in *Salmonella enterica* and *B. subtilis* ^1,9^, and is reactivated by either of the two protein deacetylases, the Zn(II)-dependent *Bs*AcuC deacetylase and/or the sirtuin CobB^10,11^. The recent researches indicated that intracellular AcP plays a critical role in a chemical acetylation reaction, can function directly as an acetyl donor^4,5^. Previous work also showed that the abundant acetyl-CoA in mitochondria (their acetyl-CoA concentration is estimated to be 0.1-1.5 mM)^12^ results in substantial spontaneous mitochondrial protein lysine acetylation^6,13^. To determine if Ac-CoA or AcP can acetylate protein, we investigated Ac-CoA/AcP-dependent acetylation using the purified recombinant *B. subtilis* Ac-CoA synthetase (*Bs*AcsA). Ac-CoA/AcP-induced acetylation on *Bs*AcsA in a dose-dependent manner was observed by Western immunoblot analysis (Fig. 1). As shown in Fig. 1, the most obvious increases in acetylation level was first observed in the presence of concentration of AcP (1 mM) or Ac-CoA (200 μM). These concentrations are within the endogenous ranges reported for Ac-CoA, which can reach cytoplasmic concentrations of 20 to 610 μM in *E. coli* cells^14,15^, and for AcP, which is estimated to be 0.2-12 mM in *E. coli* cells^4^. The intracellular levels of Ac-CoA/AcP are comparable to the concentrations of Ac-CoA/AcP needed to acetylate proteins *in vitro*, indicating that Ac-CoA/AcP are able to acetylate chemically lysine residues *in vivo*.

**Figure 2:**
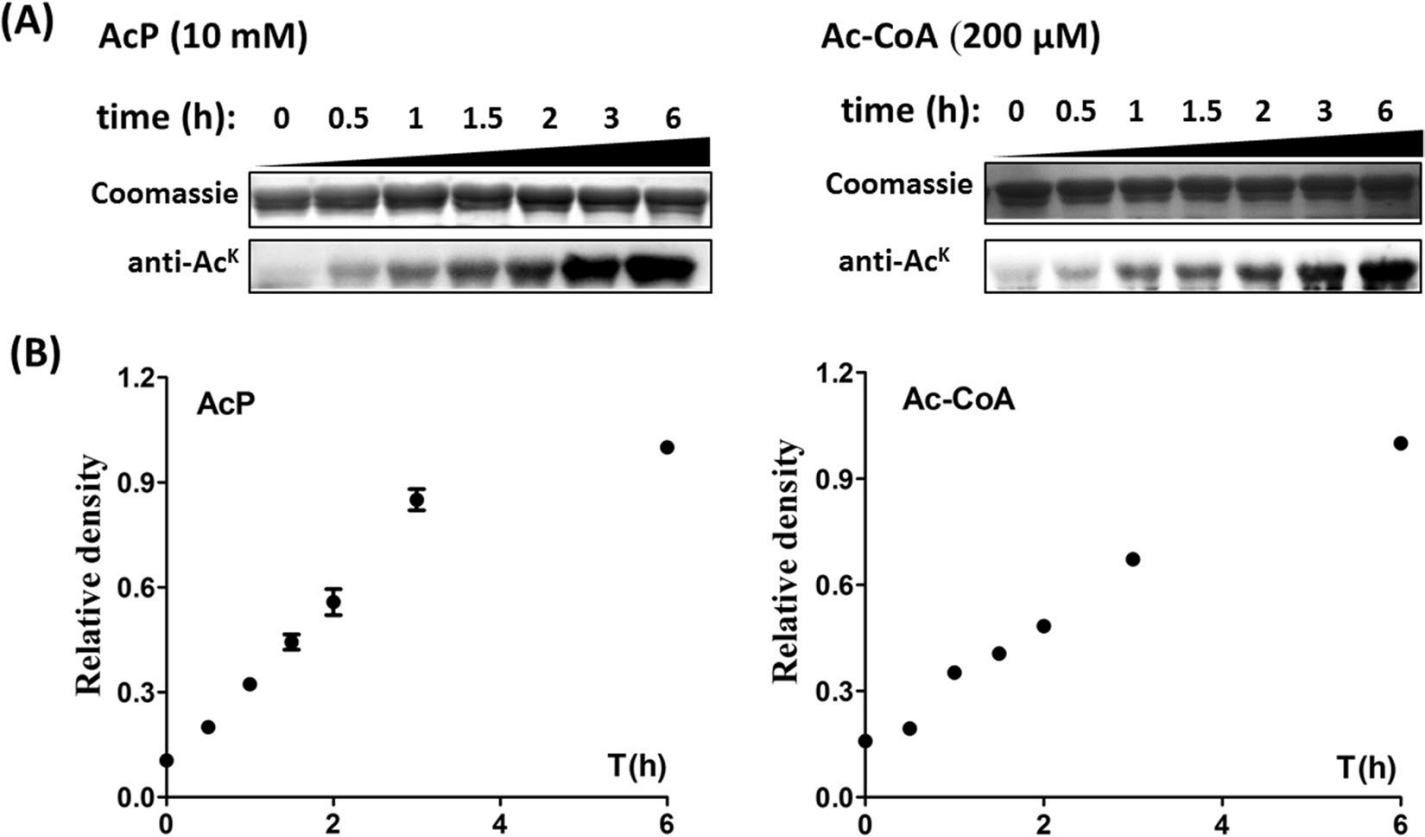
*In vitro* acetylation of *Bs*AcsA was incubated with Ac-CoA or AcP during the time of incubation. (**A**) Coomassie stain and anti acetyllysine Western blot analysis of *Bs*AcsA incubated with AcP (10 mM) or Ac-CoA (200 μM) for 0, 0.5, 1, 1.5, 2, 3, 6 h at 37 °C. (**B**) Acetylation signals were quantified using Image J software and normalized to the signal at 6 h.

The kinetic analysis of *in vitro* nonenzymatic acetylation of *Bs*AcsA was also conducted. When incubated with AcP or Ac-CoA, *Bs*AcsA became acetylated in a time-dependent manner (Fig. 2A). As shown in Fig. 2B, the results showed the Ac-CoA/AcP-dependent acetylation reaction to be slow and non-saturating, which are characteristics of non-enzymatic reactions^5^. Based on these results and previous works^4–6,16^, we conclude that Ac-CoA/AcP-dependent nonenzymatic acetylation can occur spontaneously depending on the concentrations of Ac-CoA/AcP (>1 mM AcP or 200 μM Ac-CoA).

### AcuA acetyltransferase rapidly acetylates acetyl-coenzyme A synthetase at low concentration of Ac-CoA

**Figure 3:**
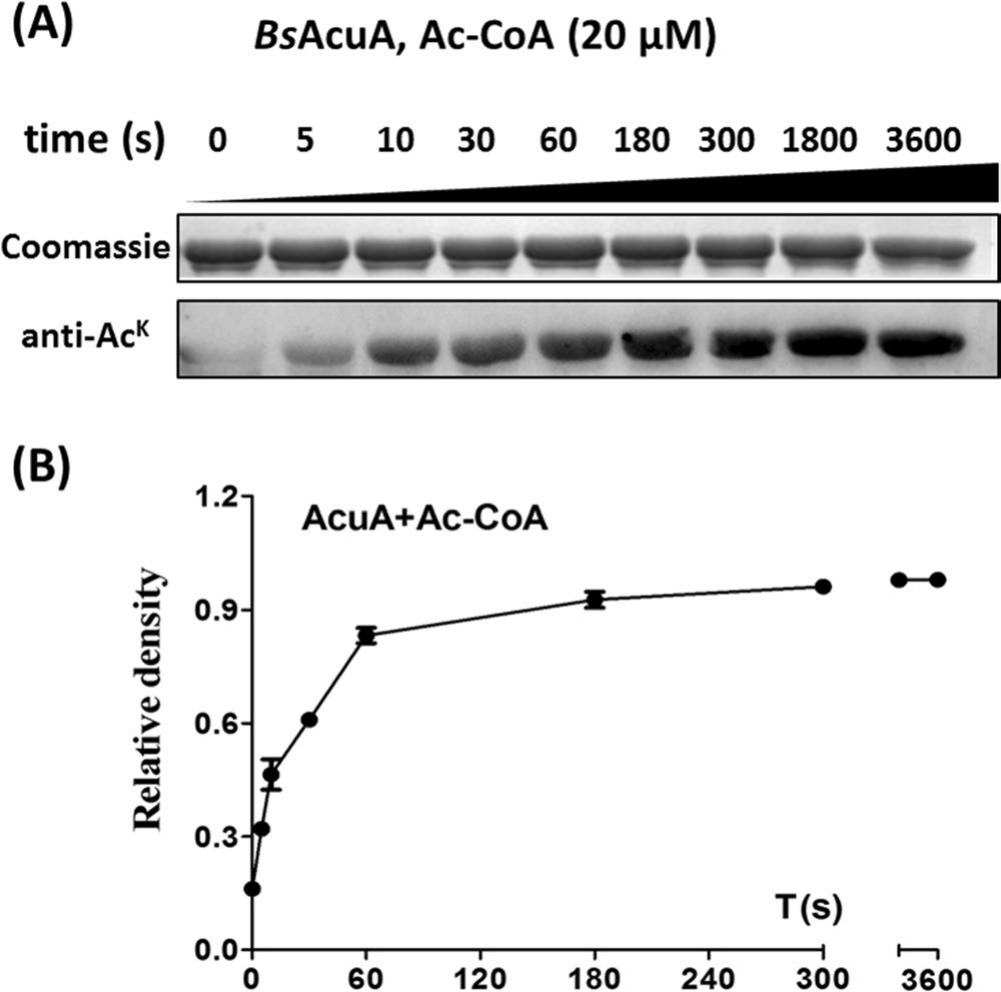
AcuA-enzymatic acetylation of *Bs*AcsA. (**A**) Coomassie stain and anti acetyllysine Western blot analysis of *Bs*AcsA incubated with Ac-CoA (20 μM) and *Bs*AcuA (10 μg) for 0, 5, 10, 30, 60, 180, 300, 1800, 3600 s at 37 °C. (**B**) Acetylation signals were quantified using Image J software and normalized to the signal at 6 h.

Previous work showed that the acetyltransferase *Bs*AcuA acetylates the conserved catalytic lysine (K549) of *Bs*AcsA^9^. Indeed, we found that *Bs*AcuA acetyltransferase quickly acetylates *Bs*AcsA at low concentration of Ac-CoA (20 μM). As shown in Fig. 3, the obvious increases in overall acetylation occurred at 5-10 s. In the absence of *Bs*AcuA, no acetylation of *Bs*AcsA was observed at the Ac-CoA concentration of <100 μM for 6 h (Fig. 1A). The kinetic analysis of AcuA-catalyzed acetylation revealed that the reaction reached saturation at 180 s. The results demonstrated that acetyltransferase *Bs*AcuA significantly accelerated the acetylation of *Bs*AcsA at low concentration of Ac-CoA (can’t chemically acetylate ε-amino group of a lysine residue).

### Acetyl-coenzyme A synthetase is inactivated by AcuA-catalyzed acetylation, but not by nonenzymatic acetylation

**Figure 4:**
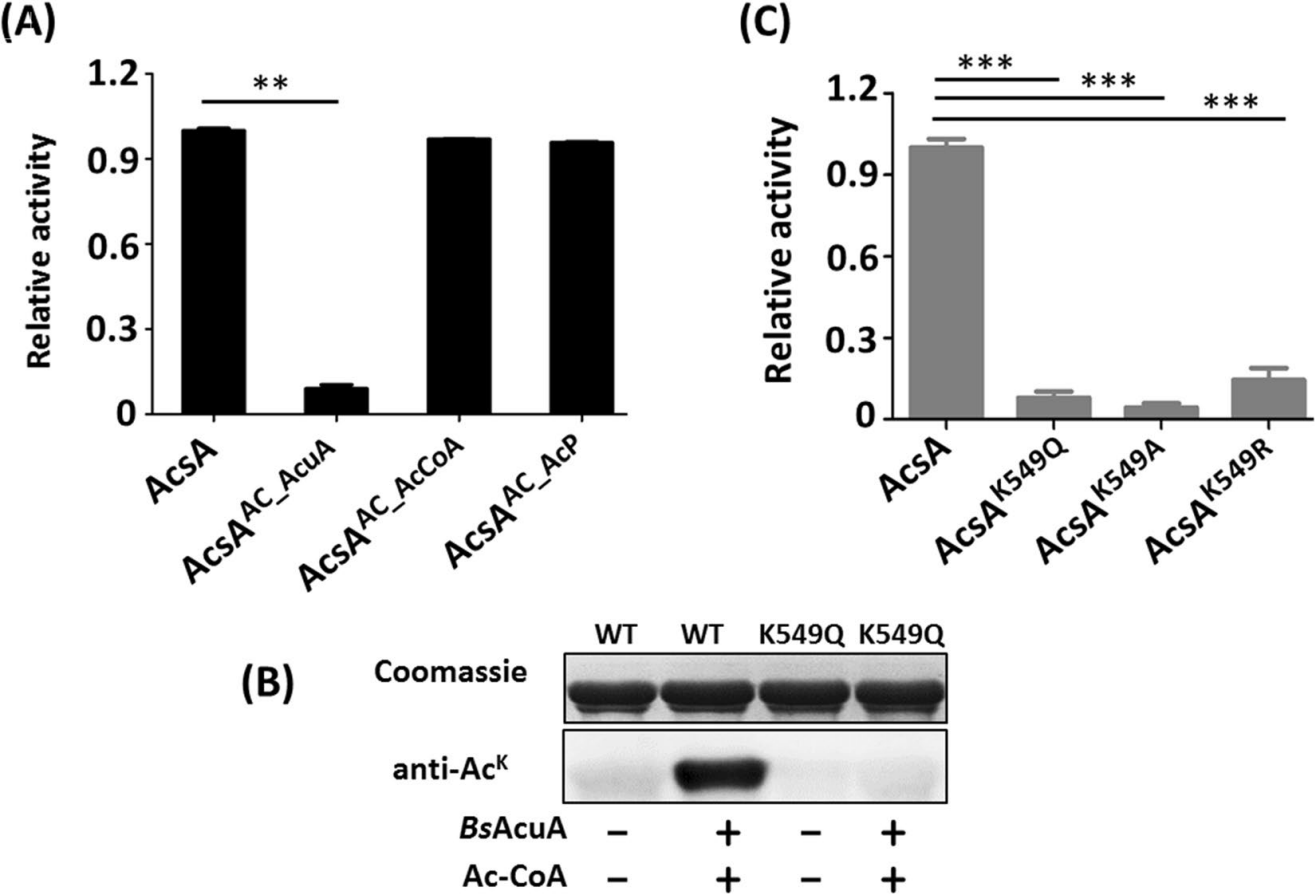
The effect of acetylation and lysine residue K549 on AcsA enzyme activity. (**A**) The effects of enzymatic and nonenzymatic acetylation on AcsA enzyme activity. (**B**) AcuA acetylated the AcsA and AcsA^K549Q^. (**C**) Activities of AcsA and its mutants (AcsA^K549Q^, AcsA^K549A^, and AcsA^K549R^). **P < 0.01, ***P < 0.001.

To investigate the effect of acetylation on AcsA enzyme activity, the activities of native (nonacetylated) and acetylated enzymes (AcuA-catalyzed acetylated AcsA, Ac-CoA/AcP-dependent acetylated AcsA) were determined. It was found that AcuA-catalyzed acetylation of Acs enzymes resulted in 85–90% decrease in acetyl-CoA synthetase activity compared to the nonacetylated enzymes, whereas Ac-CoA/AcP-dependent acetylation exerted no effect on AcsA enzyme activity (Fig. 4A). It has been reported that the conserved active-site K549 in conserved putative acylation motif PKTRSG**K** in *Bs*AcsA was acetylated by acetyltransferase *Bs*AcuA, and was critical for catalytic activity^9^. As shown in Fig. 4B, *Bs*AcuA acetylated AcsA^WT^ but did not acetylate AcsA^K549Q^, confirming that *Bs*AcuA modified only the conserved lysine residue K549 in the active site. The mutants of K549 (AcsA^K549Q^, AcsA^K549A^, and AcsA^K549R^) were essentially inactive, with 10-20% of the wild-type AcsA activity (Fig. 4C). These results indicated that residue K549 can be acetylated by AcuA-catalyzed acetylation, but not by Ac-CoA/AcP-dependent nonenzymatic acetylation. The conclusion was in agreement with the results from *Saccharopolyspora erythraea* recently reported by our group^17^. In our previous work, we found that AcP was unable to acetylate conserved lysine residue K628 of *S. erythraea* AcsA2, whereas acetylated lysine residue K611 (possible role in binding the acetate substrate), thus resulted in a 30% decrease in acetyl-CoA synthetase activity^17^. No lysine residue in *Bs*AcsA corresponding to K611 of *S. erythraea* AcsA2 was observed (Figure S1).

### Enzymatic and nonenzymatic acetylation reveal different preference for lysine sites

To determine the acetylation sites resulted from two distinct mechanisms, the acetylated *Bs*AcsA proteins (AcuA-catalyzed acetylated AcsA, Ac-CoA/AcP-dependent acetylated AcsA) were subjected to trypsin digestion, and the resulting peptides were analyzed by LTQ orbitrap Elite mass spectrometer. The data were shown in Table 1. Many acetylation sites were identified. Ac-CoA/AcP-dependent nonenzymatic mechanism acetylated more lysine residues than AcuA-catalyzed enzymatic mechanism. Surprisingly, acetylated site K549 was observed in Ac-CoA/AcP-dependent acetylated *Bs*AcsA, while some other acetylated sites besides K549 were identified in AcuA-catalyzed acetylated AcsA.

**Table 1:** The acylated sites of *Bs*AcsA under the different conditions.

Conditions		Acylated sites
Acetylation	Enzymatic (AcuA)	K16, K98, K139, K222, **K524**, K539, **K549**
	Nonenzymatic (Ac-CoA)	K16, K98, K139, K222, K524, K539, **K549**, K32, K41, K320, K346, K409, K410, K490
	Nonenzymatic (AcP)	K16, K98, K139, K222, **K524**, K539, **K549**, K32, K41, K320, K346, K409, K410, K490, K80, K164, K192, K205, K206
	*In vivo*	K16, K32, K41, K69, K77, K80, K98, K206, K320, K410, K475, K514, K516, **K524**, K539, K544, **K549**
Propionylation	Enzymatic (AcuA)	**K549**
	Nonenzymatic (Pr-CoA)	K16, K32, K41, K139, K320, K409, K514, **K524**, K539, **K549**,
	*In vivo*	K16, K32, K80, K139, K350, K409, **K524**, **K549**

**Figure 5:**
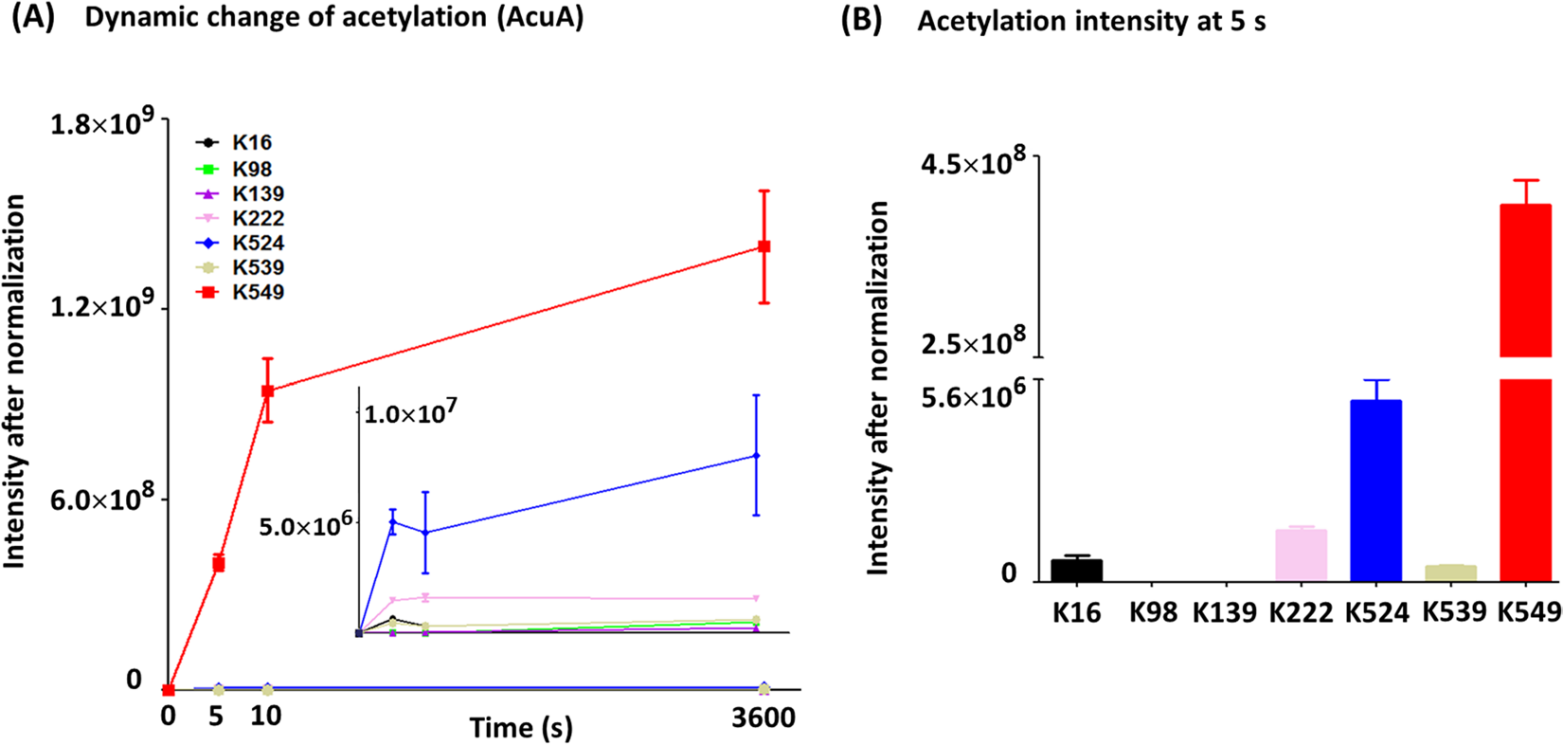
Differential AcuA-catalyzed acetylation rates at different lysine sites. (**A**) Dynamic change of acetylation during the time course. (**B**) Relative increases of acetylation at 5 s.

To obtain a more precise understanding of two distinct lysine acetylation, we monitored dynamic changes of acetylation at each lysine residue over time by label-free, quantitative mass spectrometry. It was found that lysine on the *Bs*AcsA protein exhibited different acetylation rates and different acetylation extent during the time course experiments (Fig. 5). Enzymatic and nonenzymatic acetylation reveal different preference for lysine sites with markedly different acetylation rates. The most obvious increase in acetylation was observed at K549 for AcuA-catalyzed reaction. The level of acetylation at K549 was two to three orders of magnitude higher than other acetylated sites (acetylation intensity 4.0 × 10^8^ for K549, 5.0 × 10^6^ for K524 at 5 s).

**Figure 6:**
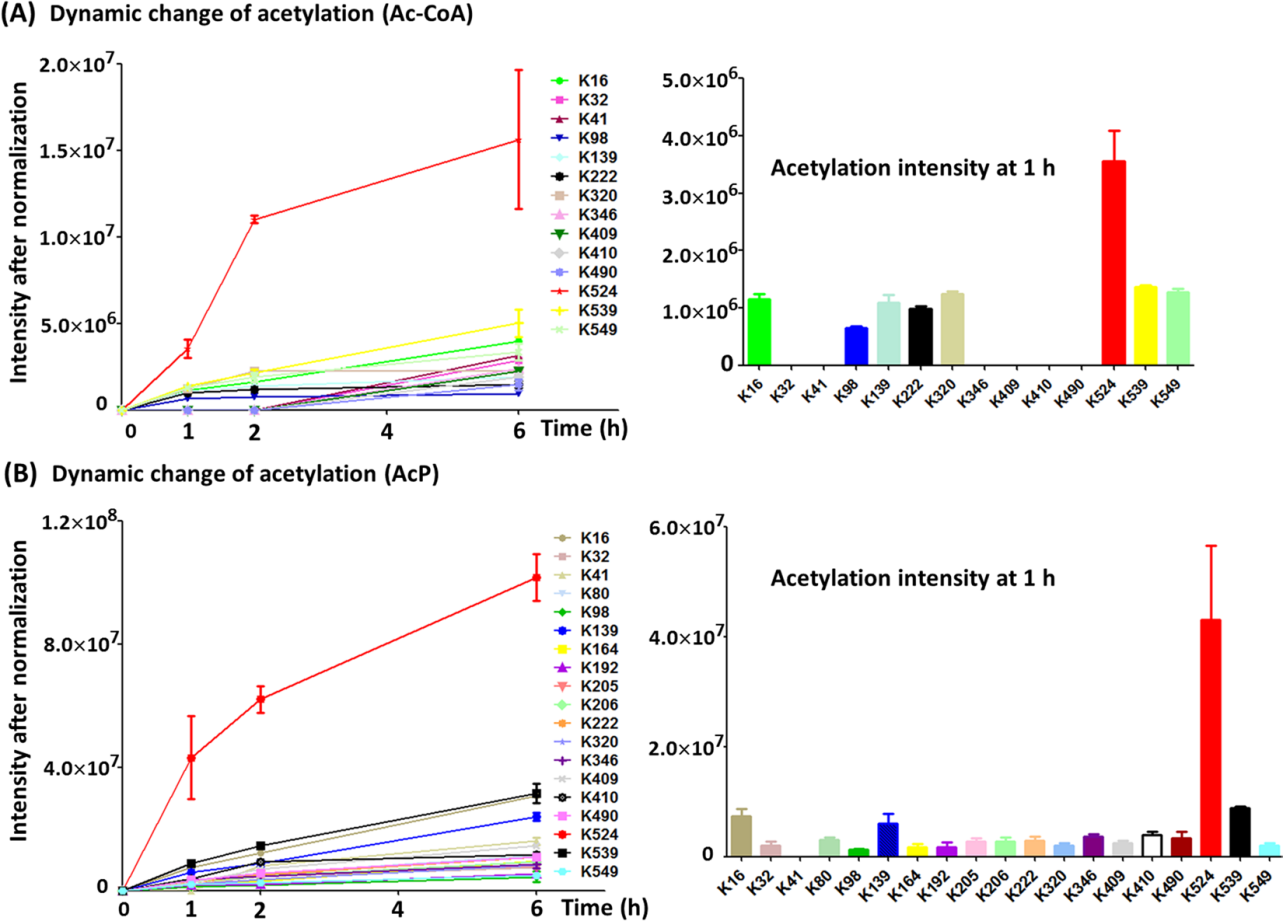
Differential nonenzymatic acetylation rates at different lysine sites. (**A**) Dynamic change of Ac-CoA-dependent acetylation during the time course and relative increases of acetylation at 1 h. (**B**) Dynamic change of AcP-dependent acetylation during the time course and relative increases of acetylation at 1 h.

**Figure 7:**
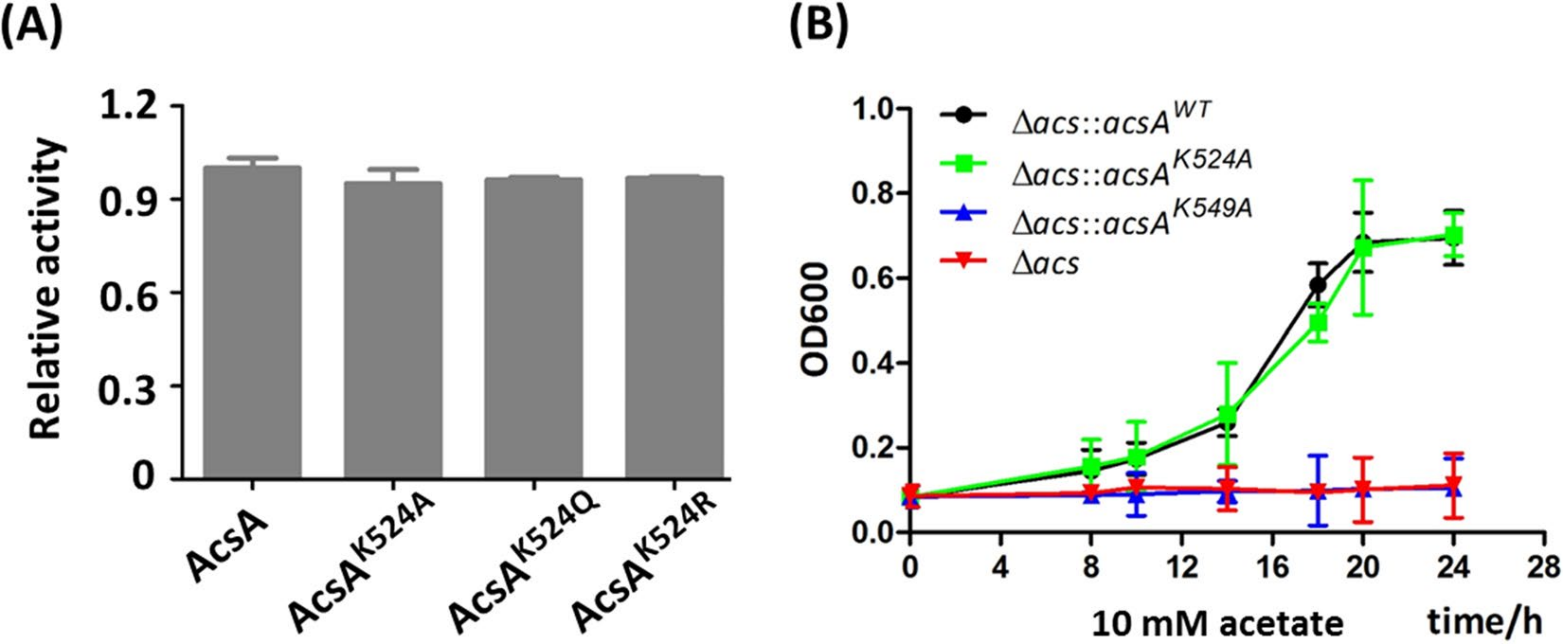
The effect of K524 and K549 on AcsA enzyme activity and growth. (**A**) Activities of AcsA and its mutants (AcsA^K524Q^, AcsA^K524A^, and AcsA^K524R^). (**B**) The growth curves of the *S. enterica* Δ*acs* strains complemented with *BsacsA* ^WT^ or mutant genes in acetate minimal medium (10 mM acetate as sole carbon source). Expression of *acs* gene was induced by addition of L-(+)-arabinose (250 µM). Cell density measurements at 600 nm were acquired. Graphed points represent the mean of three independent measurements.

A dozen acetylated lysine residues were identified on Ac-CoA/AcP-dependent nonenzymatic acetylated AcsA. As shown in Fig. 6, each lysine was acetylated to a different extent at a different rate. Among them, K524 yielded the highest reactivity with both Ac-CoA and AcP, and revealed the most significant relative increase on acetylation. Other lysines showed low reactivity. Ac-CoA/AcP-dependent acetylation of K549 occurred nonenzymatically at a low level (reflecting the low stoichiometry of acetylation), which did not impact activity of AcsA. The mutants of K524 (AcsA^K524A^, AcsA^K524Q^, and AcsA^K524R^) revealed similar activity of the wild-type AcsA, which further demonstrated that acetylation of K524 did not affect the enzymatic activity of AcsA (Fig. 7A). On the other hand, low-level acetylation of nonspecific lysine sites (not target sites of AcuA) in AcuA-catalyzed reaction perhaps resulted from Ac-CoA-dependent nonenzymatic mechanism. Indeed, these acetylated sites were similar to those detected in Ac-CoA-dependent nonenzymatic acetylated AcsA (Fig. 6A). Taken together, enzymatic and nonenzymatic acetylation revealed different preference for lysine sites (enzymatic acetylation for K549 and nonenzymatic acetylation for K524). To investigate the effects of mutation at K524 and K549 in AcsA *in vivo*, alleles encoding *B. subtilis* AcsA^WT^, AcsA^K549A^ or AcsA^K524A^ were introduced into *S. enterica* Δ*acs* strain^18^. The resulting strains were grown at 37 °C in 5 ml of the minimal acetate medium (10 mM acetate as sole carbon source). The growth was monitored in triplicate at OD600. Consistent with the activity of AcsA variants measured *in vitro* (Figs 4C and 7A), differences in growth behavior were observed. The strain synthesizing AcsA^K549A^ failed to grow on 10 mM acetate as sole carbon and energy source, while strain that synthesized AcsA^K524A^ grew as well as the AcsA^WT^ (Fig. 7B).

### Enzymatic and nonenzymatic propionylation shows similar behavior

**Figure 8:**
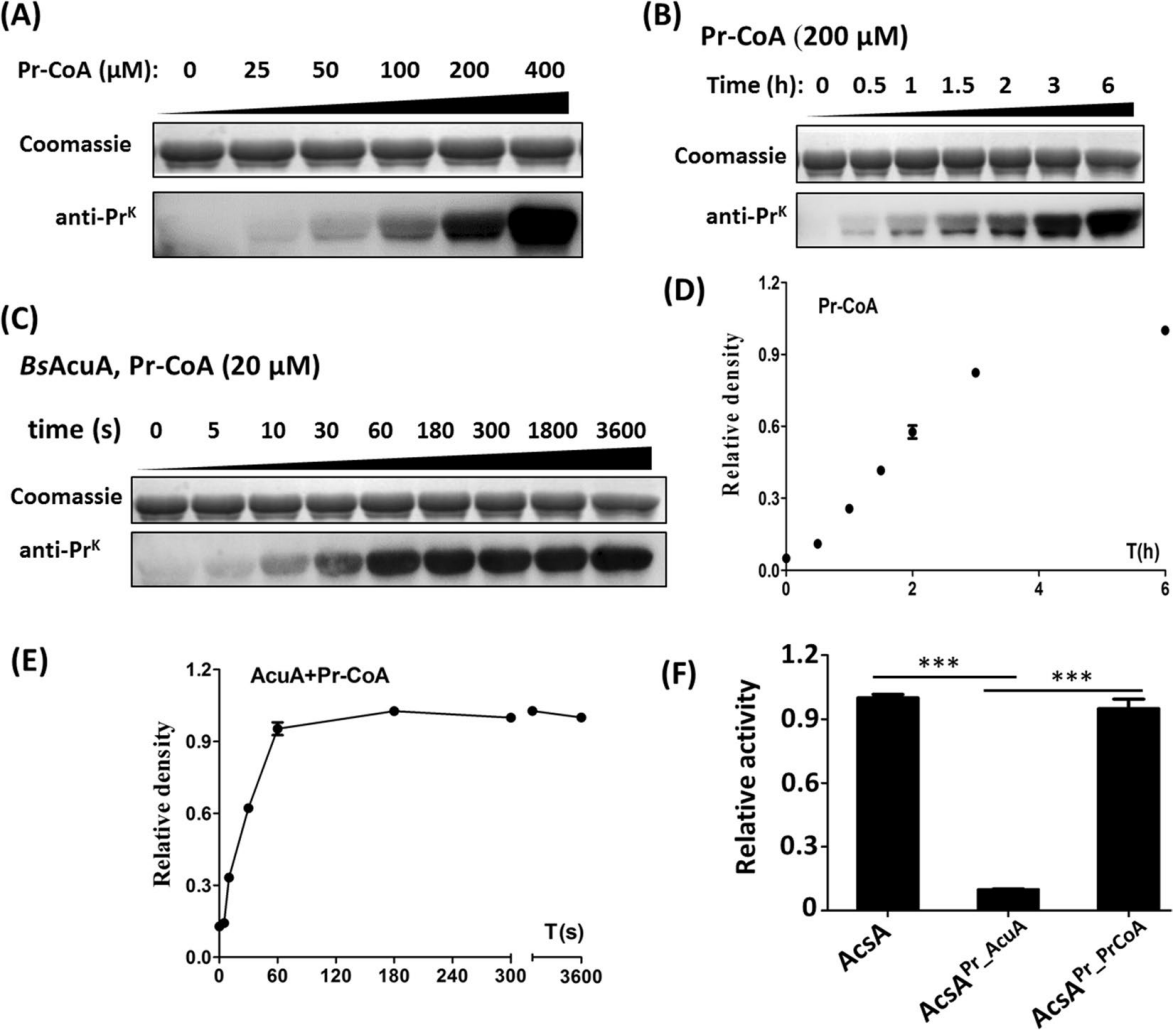
Enzymatic and nonenzymatic propionylation and their effects on AcsA enzyme activity. (**A**) Nonenzymatic propionylation of *Bs*AcsA using Pr-CoA as the propionyl group donor at the different concentrations for 6 h. (**B**) Propionylation of *Bs*AcsA was incubated with Pr-CoA (200 μM) during the time of incubation. (**C**) AcuA-catalyzed propionylation. (**D**) Propionylation signals of image (from **B**) were quantified using Image J software and normalized to the signal at 6 h. (**E**) Propionylation signals of image (from **A**) were quantified using Image J software and normalized to the signal at 6 h. (**F**) The effect of enzymatic and nonenzymatic propionylation on AcsA enzyme activity. ***P < 0.001.

**Figure 9:**
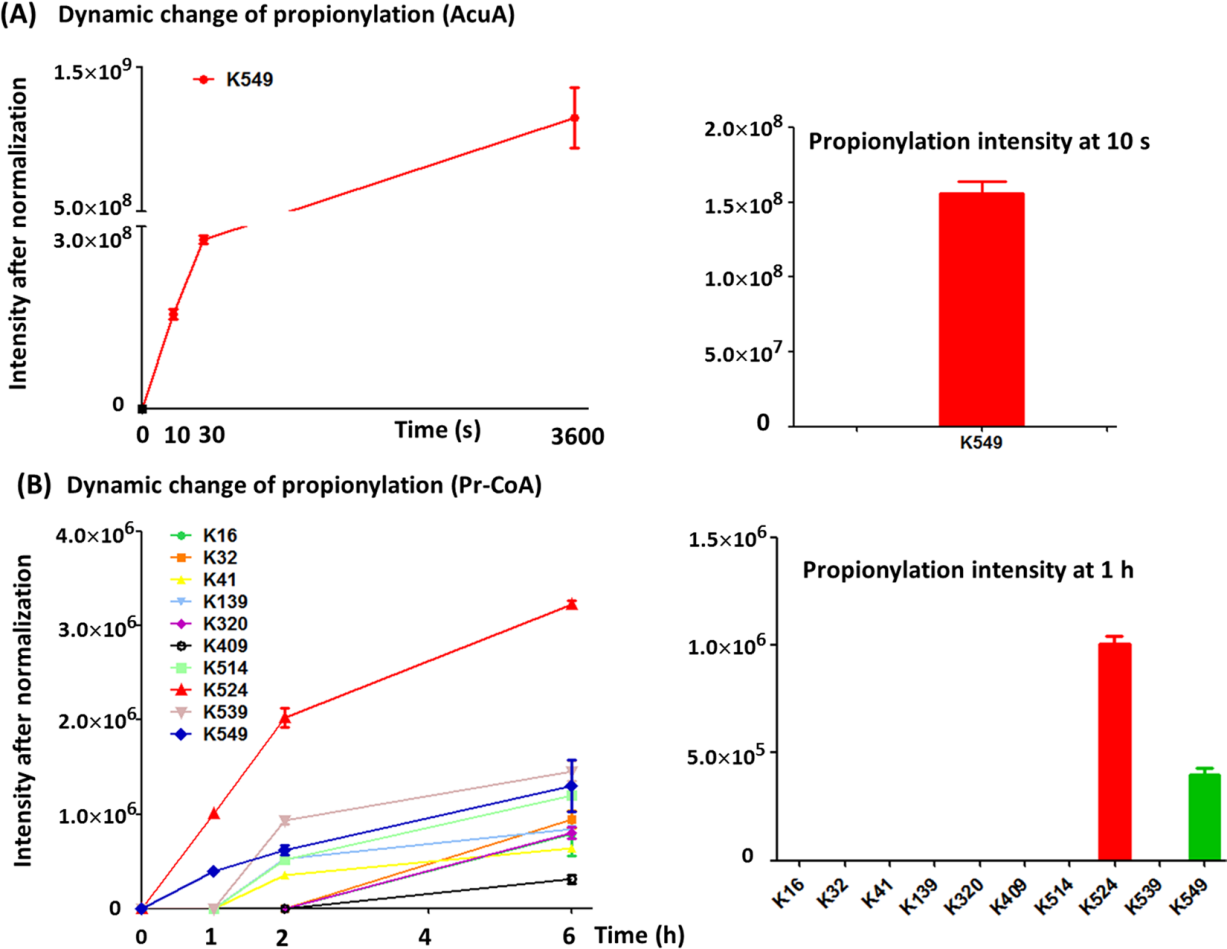
Enzymatic and nonenzymatic propionylation rates at different lysine sites. (**A**) Dynamic change of AcuA-catalyzed propionylation during the time course and relative increases of propionylation at 10 s. (**B**) Dynamic change of Ac-CoA-dependent propionylation during the time course and relative increases of propionylation at 1 h.

Enzymatic and nonenzymatic propionylation was also investigated. When incubated with propionyl-CoA, *Bs*AcsA became propionylated nonenzymatically in a dose and time-dependent manner (Fig. 8A,B). The most obvious increase in overall propionylation occurred in the presence of Pr-CoA concentration (200 μM), which far exceeds intracellular concentration of Pr-CoA 5.3 μM in *E. coli* ^15^. The result indicated that Pr-CoA-dependent nonenzymatic propionylation can’t occur spontaneously *in vivo*. Like acetylation, *Bs*AcuA acetyltransferase quickly propionylated *Bs*AcsA at low concentration of Pr-CoA (20 μM). As shown in Fig. 8C,E, the clear propionylation was observed at 30 s. The results demonstrated that acetyltransferase *Bs*AcuA can transfer propionyl group to the ε-amino group of lysines. AcuA-catalyzed propionylation inactivated AcsA, but Pr-CoA-dependent nonenzymatic propionylation not (Fig. 8F). K524 revealed the highest reactivity in Pr-CoA-dependent nonenzymatic propionylation. Two distinct mechanisms of lysine propionylation showed similar preference for lysine sites (enzymatic mechanism for K549 and nonenzymatic mechanism for K524) as acetylation (Fig. 9). The acetylation and propionylation of the two lysine sites (K524 and K549) were identified *in vivo* (Table 1).

### Structural analysis of the mechanism-sensitive lysine sites

To analyze the structural basis for selectivity, we investigated the linear neighboring amino acids of each acetyllysine, and relationship between the adjacent environment of a lysine within its folded structure and its acylation sensitivity to AcuA, AcP, or AcCoA. Two sequence logos were generated by compiling neighboring amino acids from position −10 to position +10 relative to Ac-CoA/AcP-dependent nonenzymatic acetylated lysine sites using two sample logo method^19^. As shown in Figure S2, the results revealed that to some extent acetyllysine sites tend to be position adjacent to glycine and negatively charged resides (glutamate (E) in left side and aspartate (D) in right side). However, no obvious sequence motif was observed. These appear to be consistent with the observation described by Kuhn *et al*.^5^.

**Figure 10:**
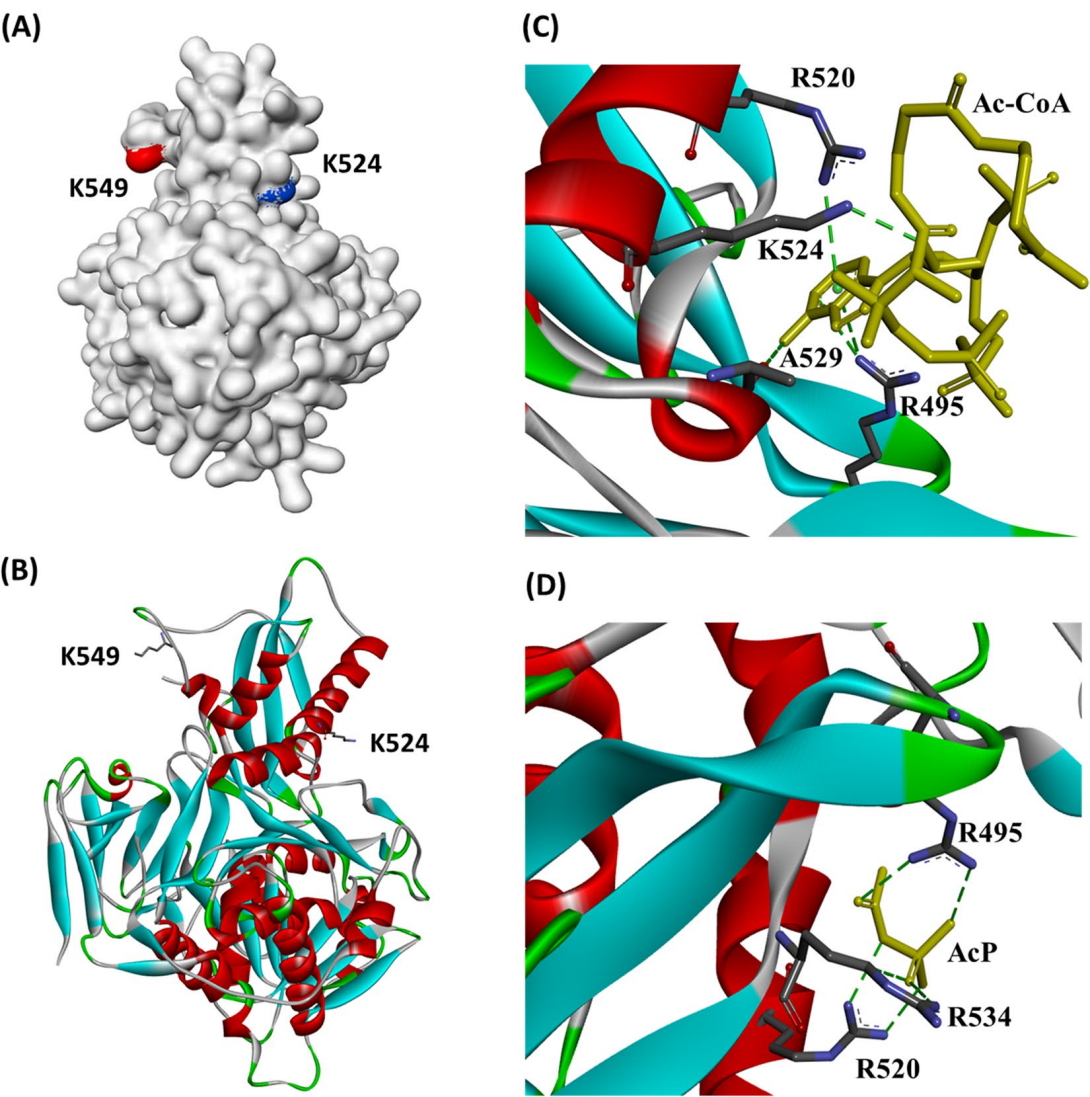
Structural analysis of the mechanism-sensitive lysine sites. (**A**) Crystal structure of *Bs*AcsA from *B. subtilis* was shown. K524 and K549 were highlighted in blue and red. (**B**) the secondary structures of *Bs*AcsA were shown. The K524 site was docked with Ac-CoA (**C**) and AcP (**D**). Green dashed lines highlight the polar contacts (Hydrogen Bond). Moleculers were displayed in yellow, nitrogen atoms are shown in light blue, carbon atoms are in grey, and oxygen atoms are in red.

The structure of *Bs*AcsA was constructed by Swiss Model server (http://swissmodel.expasy.org/) based on crystal structure of acetyl-CoA synthetase from *Salmonella enterica* (PDB 1PG4)^20^. We mapped the locations of the mechanism-sensitive lysine sites (K524 and K549) onto *Bs*AcsA structure, and analyzed the secondary structures in which K524 and K549 reside (Fig. 10). Structural analysis showed that the nonenzymatic acetylation-sensitive K524 was exposed to the surface, and the enzymatic acetylation-sensitive K549 appeared to be located near inside of the cave (Fig. 10A). It was observed that K524 was located on a stable α-helix, while K549 was on free active site loop (Fig. 10B). We further examined the amino acids adjacent to the K524 site in three-dimensional microenvironment of the folded structure, and found that positively charged arginine residues (R495, R520, and R534) were located in the vicinity of K524 (Fig. 10C,D). The charge of these arginine residues might attract negatively charged Ac-CoA and AcP. Ac-CoA and AcP molecules were coordinated to the arginine residues, and significantly acetylated K524 site but a little others.

To further evaluate the structural and microenvironmental features that affect lysine reactivity on acetylation, we determined the predicted pKa and buried ration (surface accessibility) of all lysine residues in *Bs*AcsA using Propka3.0 revision 182 (http://propka.org/)^21^. As shown in Table S1, K524 exhibited pKa value of 9.86 with buried ration of 5%, which was the smallest pKa value among the lysine residues in *Bs*AcsA with buried ration of <10%. Taken together, structural analysis showed that surface accessibility, three-dimensional microenvironment, and pKa value of K524 residue enhance the its reactivity toward AcP and Ac-CoA, and favour nonenzymatic acylation reaction.

In summary, we systematically investigated the site-specific and kinetic characterization of AcuA-catalyzed enzymatic and AcCoA/AcP-dependent nonenzymatic acetylation. *B. subtilis* acetyl-CoA synthetase (*Bs*AcsA) can be acetylated *in vitro* either catalytically by lysine acetyltransferase *Bs*AcuA and Ac-CoA (at low concentration), or nonenzymatically by Ac-CoA or AcP (at high concentration). Acetyl-CoA synthetase is inactivated by AcuA-catalyzed acetylation, but not by nonenzymatic acetylation. The results showed that lysine residues on the same protein exhibit different acetylation reactivity with acetyl-phosphate and acetyl-CoA, which was determined by surface accessibility, three-dimensional microenvironment, and pKa value of lysine. The enzymatic and nonenzymatic acetylation revealed different preference for lysine sites (enzymatic acetylation for K549 and nonenzymatic acetylation for K524).

## Materials and Methods

### Bacterial strains, growth conditions, and chemicals

The strains and plasmids used in this study were listed in Table 2. *Bacillus subtilis* 168 and *E. coli* were grown on Luria-Bertani (LB) medium. When needed, antibiotics were added to the medium at the following concentrations: ampicillin, 100 μg/ml; kanamycin, 50 μg/ml. All media were sterilized by autoclaving at 121 °C for 20 min.

**Table 2:** The strains and plasmids used in this study.

Strain or plasmid	Source or reference
strains	
*Bacillus subtilis* 168	ATCC 6051
*E. coli* DH5α	TransGen Biotech
*E. coli* BL21(DE3)	TransGen Biotech
*E. coli* BL21(DE3)-*Bs*AcsA	In this work
*E. coli* BL21(DE3)-*Bs*AcuA	In this work
*Salmonella enterica* Δ*acs (JE7758)*	^18^
plasmids	
pET-28a	Thermo Scientific
pBAD30	^18^
pGEX-4T-2	Thermo Scientific
pET-*Bs*AcsA	Lab stock
pGEX-*Bs*AcuA	In this work

The antibodies and columns for purification of proteins used were as follows: anti-acetyl-lysine antibody (ImmuneChem Pharmaceuticals, Burnaby, CA); pan anti-propionyl-lysine antibody (PTM Biolabs); HRP conjugates (anti-PrK; ImmuneChem); glutathione transferase (GST) agarose column and nickel-nitrilotriacetic acid (Ni-NTA) superflow column (Qiangen, Valencia, CA).

### Overproduction and purification of proteins

The genes for heterologous expression of protein acetyltransferase *Bs*AcuA and acetyl-CoA synthetase *Bs*AcsA were amplified by PCR from template DNA of *B. subtilis* 168, and proteins were expressed using the *E. coli* BL21 (DE3) strain. The primers used in this study are listed in Table S2. The gene coding for AcsA was inserted into the the EcoRI and HindIII sites of plasmid pET-28, and *acuA* gene was cloned into pGEX-4T-2 that was digested by EcoRI and NotI by one step cloning kit (Novoprotein Scientific, Summit, NJ) respectively, in order to generate a 6-His tag or GST-tag fusion protein. These constructed *E. coli* BL21(DE3) strains were grown overnight in 5 ml LB medium, and then cultures were transferred into 100 ml LB medium at 37 °C supplemented with kanamycin or ampicillin in shaking flasks. Expression of the cloned genes was induced by the addition of IPTG (0.4 mM), followed by overnight incubation at 20 °C.

Cells were harvested by centrifugation and washed twice wtih 20 mM ice-cold PBS buffer (pH 8.0), resuspended with PBS, then broken by sonication on ice using Ultrasonic cell crusher. Shortly thereafter, cell debris was removed by centrifugation 4 °C. His-tagged proteins were purified using Ni-NTA Superflow columns (Qiangen, Valencia, CA). And bound proteins were eluted using a linear gradient from 20 to 250 mM imidazole 50 mM NaH_2_PO_4_ and 300 mM NaCl (pH 8.0). In addition, GST-AcuA was purified by GST affinity chromatography as previously described^9^. Next, these purified proteins were analyzed by SDS-PAGE. Protein concentration was monitored by the BCA method using buffer PBS as the control and the standard curve was determined by BSA.

### *In vitro* Acyl-CoA synthetase assays

The activity of acetyl-CoA synthetase were determined as previously reported^22,23^. Briefly, the reaction mixture (100 μl) included 2 mM of acyl substrates (sodium acetate or sodium propionate), 5 mM ATP, 3 mM MgCl_2_, 0.5 mM dithiothreitol, CoA (2.5 mM), 300 mM hydroxylamine and 50 mM Tris-HCl buffer (pH 8.0). The assay was initiated by adding suitable amount of enzyme, and the reaction mixture was incubated at 37°C for 20 min. The reaction was terminated by adding 100 μl of 2.5% (w/v) FeCl_3_ in 2 M HCl containing 10% perchloric acid. The denatured protein was removed by centrifugation at 9000 rpm for 5 min, and the absorbance at 520 nm was measured. The standard curves for acyl-CoA were prepared for estimating the enzyme activity, and every sample was averaged from three independent determinations.

### Western blot analysis

Protein samples were separated by SDS-PAGE and transferred to a PVDF membrane (Merck, Millipore). The PVDF membrane was blocked at room temperature for 2 h in BSA Blocking Buffer Western Blot I (BSA) (CWBIO, China). After washing with TBST buffer (20 mM Tris-HCl, pH 7.6, 150 mM NaCl, and 0.1%(v/v) Tween 20) three times, the membrane (Millipore) was incubated with anti-acetyl-lysine antibody (Anti-acK) or anti-propionyl-lysine (Anti-prK) antibody which was diluted 1:1000 in TBST at 4 °C for overnight. Then after washing with TBST, the Anti-prK membrane incubated with HRP-conjugated goat anti-rabbit secondary antibody at 1:10000 dilution in TBST for 1 h at room temperature. Eventually, signal detection was tested by ECL system (CTB, USA) according to the manufacturer.

### Site-directed mutagenesis of *Bs*AcsA acetylated site

Site-directed mutations at K549 or K524 of *Bs*AcsA were inserted into the pET28a plasmid using the fast mutagenesis system (Transgen Biotech, China) as described previously^23^. All site-directed genes were amplified by PCR from the template DNA of *B. subtilis* 168 with the primers listed in Table S2. Next, the DMT enzyme (Transgen Biotech, China) can be directly added to the PCR products systems, and the reaction mixtures were inserted into the EcoRI and HindIII sites of plasmid pET-28 for expression as His tagged fusion proteins by one step cloning kit (Novoprotein Scientific). In order to express protein, these plasmids were transformed into the *E. coli* BL21 (DE3) strain.

### Enzyme-dependent protein acetylation and propionylation

AcuA-catalyzed acetylation and propionylation were carried out as reported^22,24^. Briefly, purified *Bs*AcsA (10 μg) was incubated at 37 °C with GST-H6-AcuA (10 μg) and 20 μM Pr-CoA (Sigma) or Ac-CoA (20 μM) in HEPES-NaOH buffer (50 mM, pH 7.5) containing tris(2-carboxyethyl) phosphine hydrochloride (200 μM); final volume was 200 µl. After 2 h, samples were divided into two portions for SDS-PAGE and Western blot, meanwhile the other was used for measurement of activity.

### ***In vitro*** nonenzymatic acetylation and propionylation

Purified *Bs*AcsA (10 μg) were incubated at 37 °C with AcP, Ac-CoA or Pr-CoA (Sigma) in Tris-HCl (50 mM, pH 8.0) containing NaCl (150 mM) for 6 h^6,13^. After the acylation reaction, samples were analyzed by SDS-PAGE, Western blot, and measurement of the activity.

### Identification of *in vivo* acetylation and propionylation

*Bacillus subtilis* AcsA^WT^ was introduced into *S. enterica* Δ*acs* strain^18^. The resulting strain was grown at 37 °C in the minimal medium (acetate or propionate as sole carbon source). The induced endogenous *Bs*AcsA^WT^ enzymes in *S. enterica* Δ*acs* strains were analyzed by mass spectrometry for identification of acetylated sites (in acetate minimal medium) and propionylated sites (in propionate minimal medium).

### In Gel Digestion and Label-Free Quantification

For sample preparation, gels were excised from the SDS-PAGE gels, then destained with 50% ethanol. After that, the gels were sliced into small pieces and dehydrated with 100% acetonitrile. The proteins in gels were reduced by 10 mM DTT at 56°C for 40 min and alkylated by 15 mM iodoacetamide in darkness for 45 min at room temperature. 50% acetonitrile / 50% 50 mM ammonium bicarbonate (v/v) were used to remove the reagents used and proteins underwent digestion by using trypsin (an enzyme-to-substrate ratio of 1:30). Digested peptides were extracted from the gels by with 50% ACN/50% 0.1% trifluoroacetic acid (v/v) for 30 min and then 100% ACN for 10 min. Peptides were dried in a SpeedVac (Thermo Fisher Scientific, Waltham, MA) before LC-MS/MS analysis^17,25^.

Peptide samples dissolved in solvent A (0.1% formic acid and 2% acetonitrile in water) were injected onto a reverse-phase C18 column (18 cm length × 75 μm inner diameter; C18 resin with 3 μm particle size; 90 Å pore diameter; Dikma Technologies Inc., Lake Forest, CA) coupled to a nano-HPLC system (Thermo Fisher Scientific, Waltham, MA) and eluted by 60 min gradient with 8−30% solvent B (0.1% formic acid and 10% water in acetonitrile) for 51 min, 30–48% solvent B for 5 min, 48–80% solvent B for 1 min, and 80% solvent B for 3 min at a flow rate of 300 nL/min. LTQ orbitrap Elite mass spectrometer (Thermo Fisher Scientific) was used for analysis with the full MS spectra with an m/z range of 350 to 1700. A mass resolution of 120000 at m/z 200 was set and 15 most intense ions were sequentially isolated for MS/MS fragmentation by using collision-induced dissociation with a normalized collision energy of 35%. Ions with either a single charge or more than four charges were discard. The electrospray voltage was set at 2 kV and dynamic exclusion duration was maintained as 40 s.

Raw data was converted to mgf files by using Thermo Proteome Discoverer v1.4.0.288, then the mgf files were searched against the *Bacillus subtilis* database with a Mascot search engine. The search parameters were: enzyme, trypsin/P; missed cleavage, 2; fixed modification, carbamidomethy (C); variable modification, acetyl (protein N-term), oxidation (M) and propionyl or acetyl (K); 10 ppm for MS and 0.5 Da for MS/MS. Qualitative and quantitative analysis were conducted for propionyl or acetyl sites. Areas under the curves (AUC) of the precursor ion’s peak extracted from the ion chromatograms was used to evaluate the MS intensity^26^. Data were presented by using the normalized intensity. We normalized the protein level by using the peak area of the unmodified peptide ‘HVLSVGEPLNPEVIR’ in each sample. All samples were performed on two replicates.

### pKa Calculations and surface accessibility

pKa values and buried ratio of *Bs*AcuA were calculated using propka3.0 (revision 182) (http://propka.org/). And propka calculated the surface accessibility, defining a parameter referred to as buried ratio^5,21^.

## Electronic supplementary material

Table S1-S2, Figure S1-S9
