# Supplementary material for: Site-specific and kinetic characterization of enzymatic and nonenzymatic protein acetylation in bacteria
Source: Sci Rep. 2017 Nov 1;7:14790. doi: 10.1038/s41598-017-13897-w (PMC5665961; doi:10.1038/s41598-017-13897-w)
Supplement: Supplementary file 1 — Table S1-S2, Figure S1-S9 [file 41598_2017_13897_MOESM1_ESM.pdf]

Site-specific and kinetic characterization of enzymatic and nonenzymatic protein acetylation in bacteria

Miao-Miao Wang[1,2§], Di You[2§], Bang-Ce Ye*[1,2]

[1]Collaborative Innovation Center of Yangtze River Delta Region Green Pharmaceuticals, College of Pharmaceutical Sciences, Zhejiang University of Technology, Hangzhou 310014, Zhejiang, China
[2]Lab of Biosystems and Microanalysis, State Key Laboratory of Bioreactor Engineering, East China University of Science and Technology, Shanghai, 200237, China

[§]These authors contributed equally to this work.

To whom correspondence should be addressed:

Bang-Ce Ye
Professor, College of Pharmaceutical Sciences
Zhejiang University of Technology, Hangzhou 310014, Zhejiang, China
Tel/Fax: 0086-21-64252094
Email: yebangce@zjut.edu.cn; bcye@ecust.edu.cn

Supporting Information

Table S1. The pKa and buried ratio of lysine residues in *Bs*AcsA

Residue	pKa	Buried	Residue	pKa	Buried	Residue	pKa	Buried
**Lys32**	10.30	23%	**Lys164**	10.85	31%	**Lys410**	11.04	0%
**Lys41**	10.45	0%	**Lys192**	10.57	0%	**Lys425**	10.26	0%
**Lys58**	10.46	0%	**Lys205**	9.40	38%	**Lys475**	10.50	50%
**Lys60**	11.14	13%	**Lys206**	10.59	17%	**Lys490**	10.23	4%
**Lys66**	8.45	60%	**Lys222**	9.19	24%	**Lys500**	11.74	37%
**Lys69**	10.47	0%	**Lys239**	11.19	1%	**Lys514**	10.58	0%
**Lys73**	10.73	3%	**Lys245**	10.53	0%	**Lys516**	10.40	0%
**Lys77**	10.59	0%	**Lys320**	10.59	0%	**Lys524**	9.86	5%
**Lys80**	10.98	35%	**Lys346**	10.35	0%	**Lys539**	9.08	26%
**Lys98**	11.12	18%	**Lys350**	10.31	0%	**Lys541**	10.46	0%
**Lys121**	7.53	88%	**Lys375**	11.56	0%	**Lys544**	11.29	0%
**Lys139**	10.49	0%	**Lys381**	10.62	0%	**Lys549**	10.33	0%
**Lys148**	10.67	0%	**Lys409**	11.45	0%	**Lys556**	6.86	76%

Table S2. Primers for overproduction of proteins and mutations

Primers	Sequence (5'-3')
BSU29680	ATGGGTCGCGGATCCGAATTCATGAACTTGAAAGCGTTACCAGC
	CTCGAGTGCGGCCGCAAGCTTTTAATCCTCCATTGTTGACAGATC
BSU29690 (GST)	CGCGTGGATCCCCAGGAATTCGTGGAACATCATAAAACATACC
	AGTCAGTCACGATGCGGCCGCTAATACATATAACGATGATAAAAA
pET-28-T	TAATACGACTCACTATAGGG
	TGCTAGTTATTGCTCAGCGG
pGEX-4T-T	GGCTGGCAAGCCACGTTTGGTG
	CCGGGAGCTGCATGTGTCAGAGG
K524Q	AAGAGATCCGCCTATTTGTACAGCAGGGTCTTGC
	GTACAAATAGGCGGATCTCTTCTTTCAGTTTATCAG
K524R	AAGAGATCCGCCTATTTGTAAGGCAGGGTCTTGC
	CTTACAAATAGGCGGATCTCTTCTTTCAGTTTATCAG
K524A	AAGAGATCCGCCTATTTGTAGCGCAGGGTCTTGC
	GCTACAAATAGGCGGATCTCTTCTTTCAGTTTATCAG
K549Q	CTTCCGAAAACCAGAAGCGGACAGATCATGAGGCGCGTG
	GTCCGCTTCTGGTTTTCGGAAGCTTATCTTTAAATTCGATCTCAC
K549R	CTTCCGAAAACCAGAAGCGGAAGGATCATGAGGCGCGTG
	CTTCCGCTTCTGGTTTTCGGAAGCTTATCTTTAAATTCGATCTCAC
K549A	CTTCCGAAAACCAGAAGCGGAGCGATCATGAGGCGCGTG
	GCTCCGCTTCTGGTTTTCGGAAGCTTATCTTTAAATTCGATCTCAC

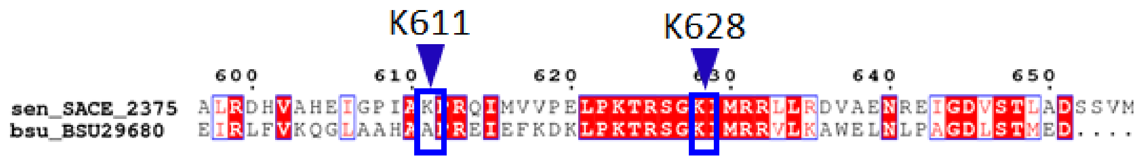

Figure S1. No lysine residue in *Bs*AcsA (BSU29680) corresponding to K611 of *S. erythraea* AcsA2 (SACE_2375) was observed

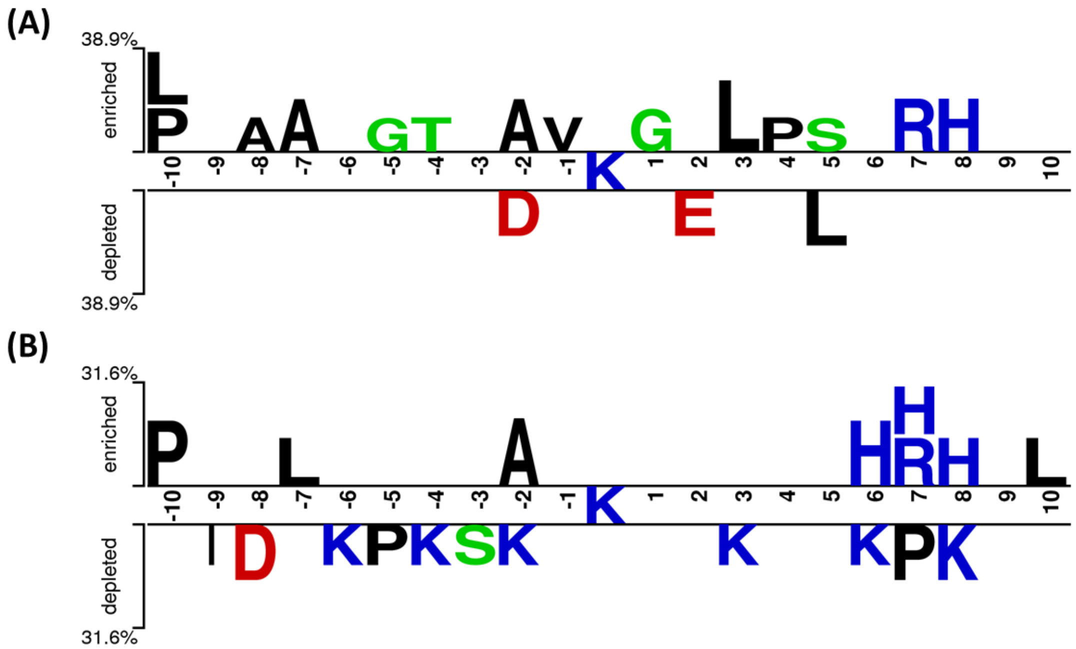

Figure S2. Analysis of the amino acid composition and position relative to acetyllysines. (A) A two sample logo was generated showing the amino acid composition in positions -10 to +10 relative to 14 lysines in Ac-CoA-dependent nonenzymatic acetylation. Only amino acid residues significantly enriched and depleted (0.05< P < 0.1; t-test) are shown. (B) A two sample logo was generated showing the amino acid composition in positions -10 to +10 relative to 19 lysines in AcP-dependent nonenzymatic acetylation. Only amino acid residues significantly enriched and depleted (0.05< P < 0.1; t-test) are shown.

Raw mass spectrometry data

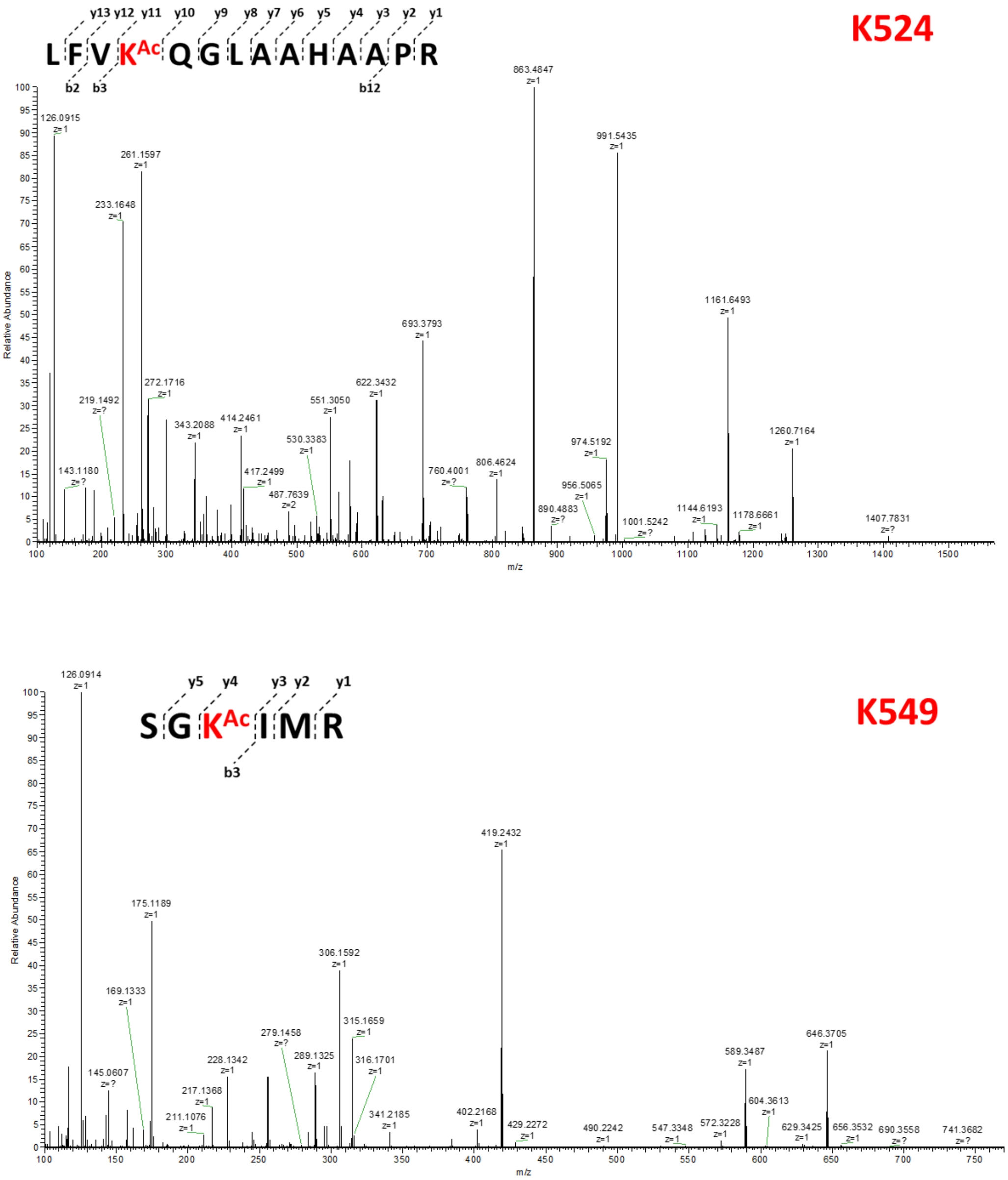

Figure S3. The MS/MS spectra of acetylpeptides containing K524 and K549 from the *Bs*AcsA^{WT} protein isolated from strain grew on acetate.

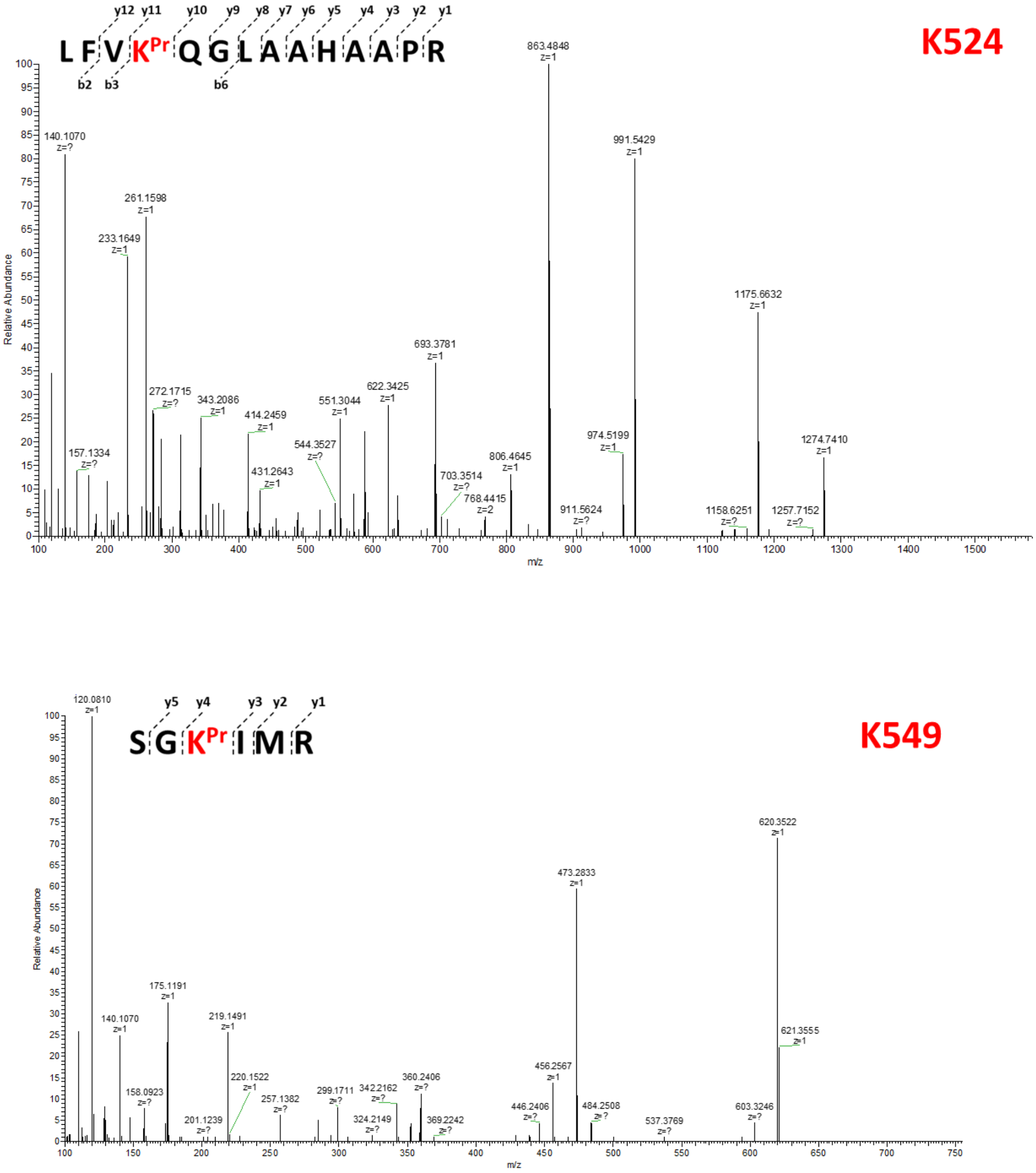

Figure S4. The MS/MS spectra of propionylpeptides containing K524 and K549 from the *Bs*AcsA^{WT} protein isolated from strain grew on propionate.

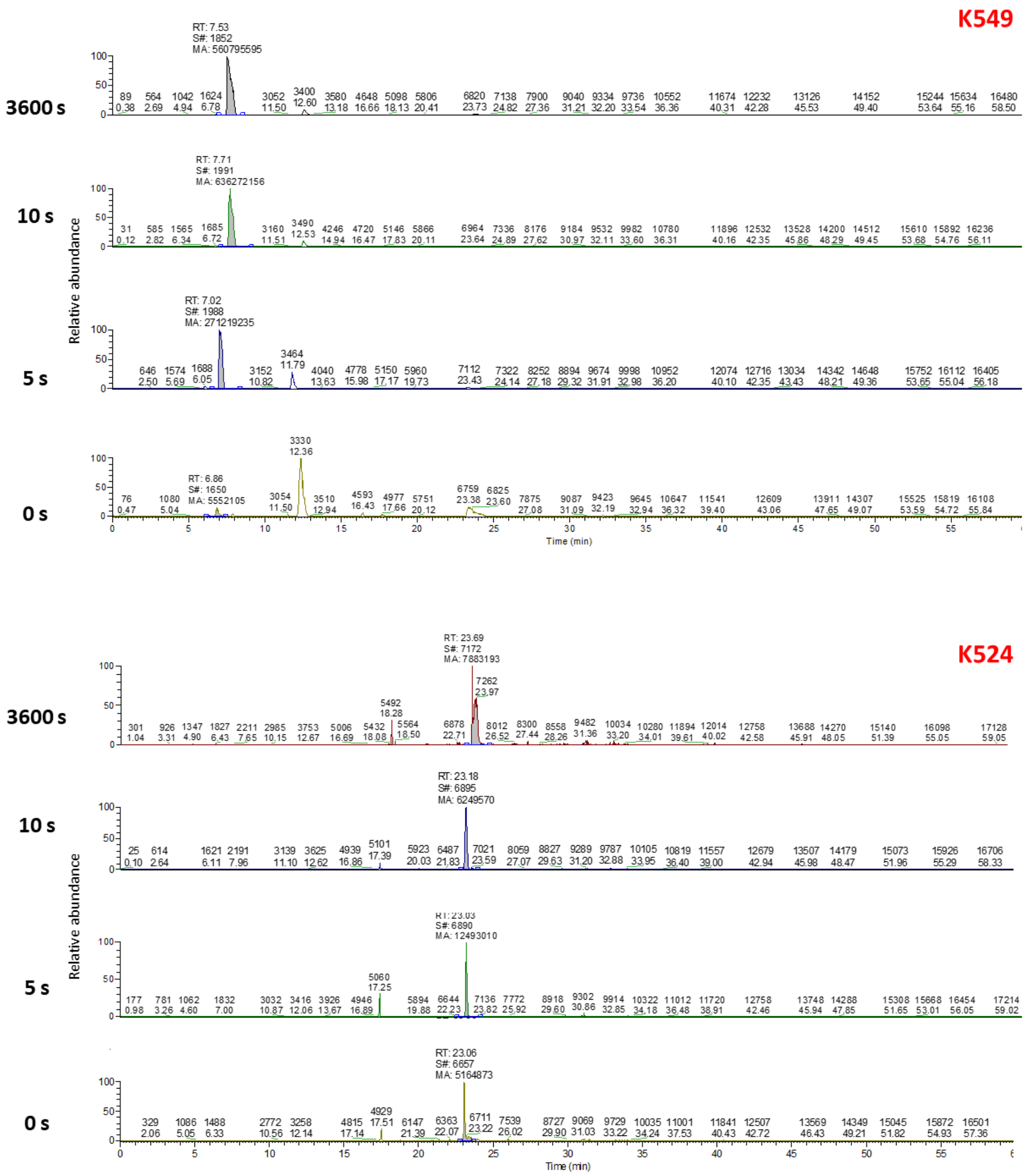

Figure S5. Mass spectrometry quantitative analysis of the AcuA-acetylated peptides containing K524 and K549. The effective AUC (areas under the curves) was in gray.

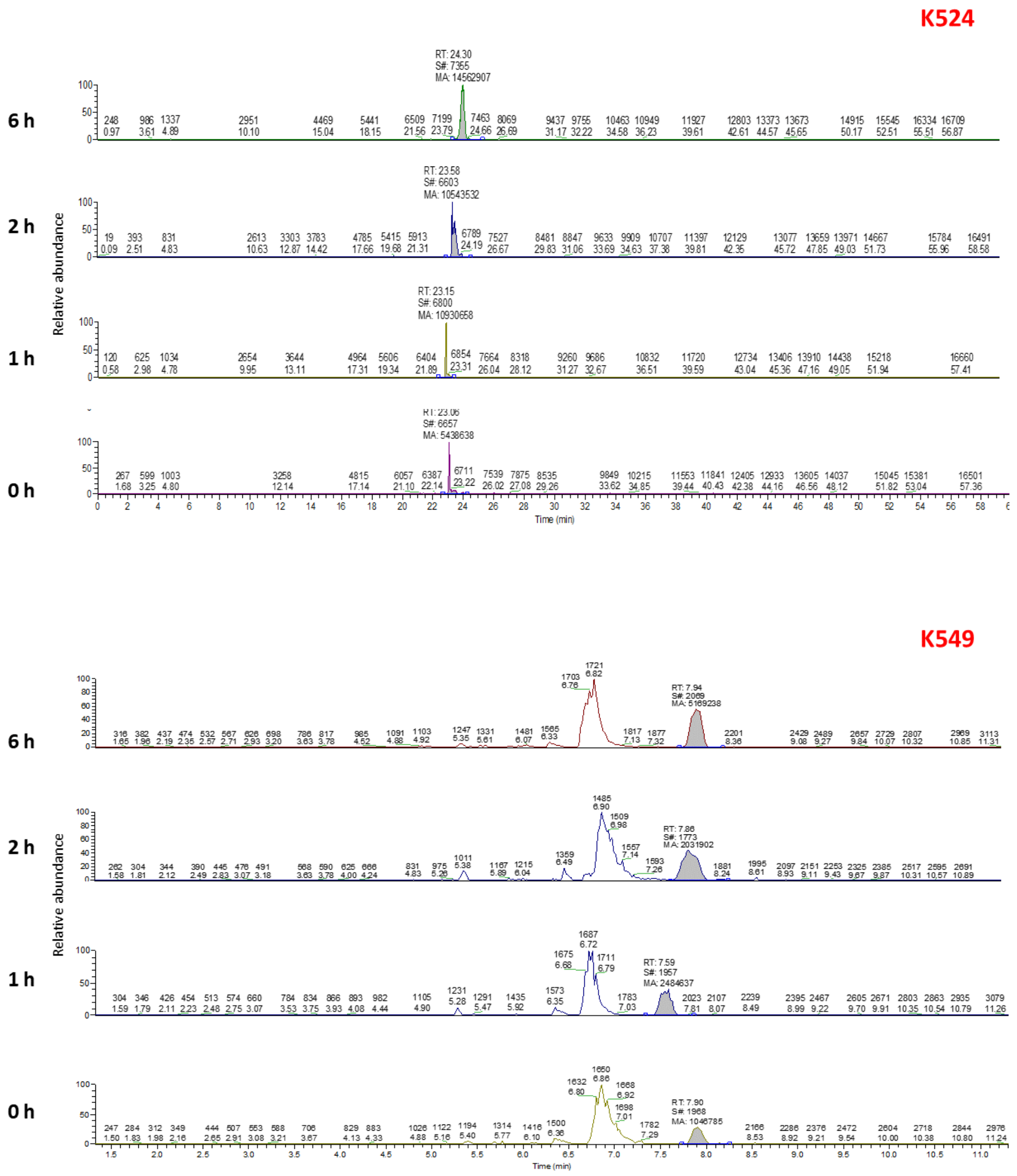

Figure S6. Mass spectrometry quantitative analysis of the Ac-CoA-acetylated peptides containing K524 and K549. The effective AUC (areas under the curves) was in gray.

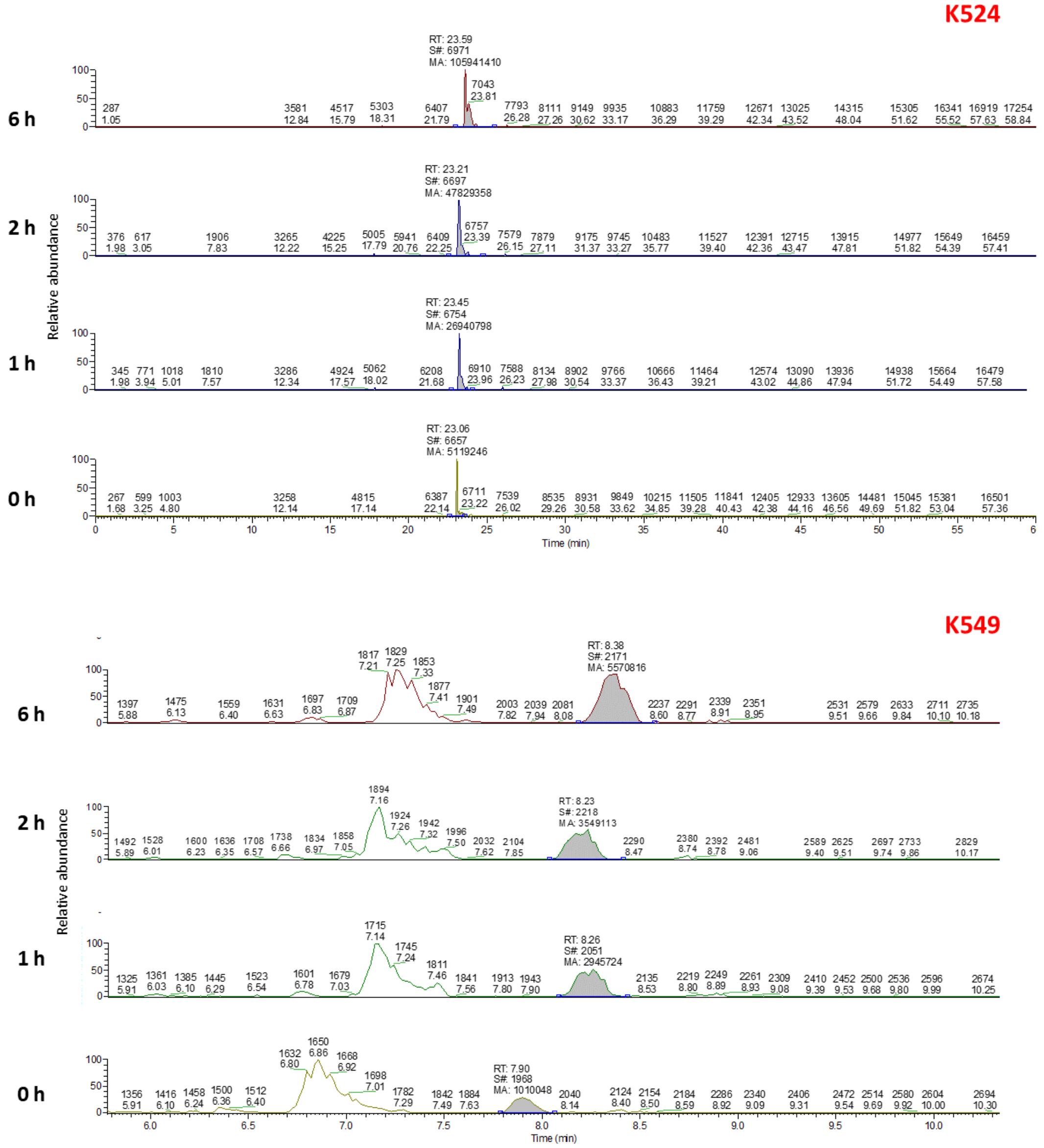

Figure S7. Mass spectrometry quantitative analysis of the AcP-acetylated peptides containing K524 and K549. The effective AUC (areas under the curves) was in gray.

K549

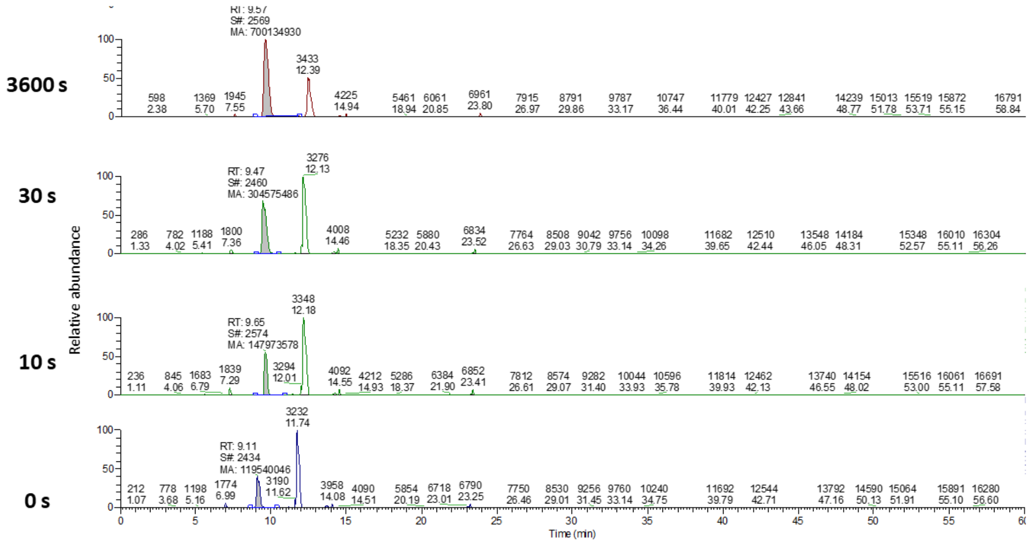

Figure S8. Mass spectrometry quantitative analysis of the AcuA-propionylated peptides containing K549. The effective AUC (areas under the curves) was in gray.

K524

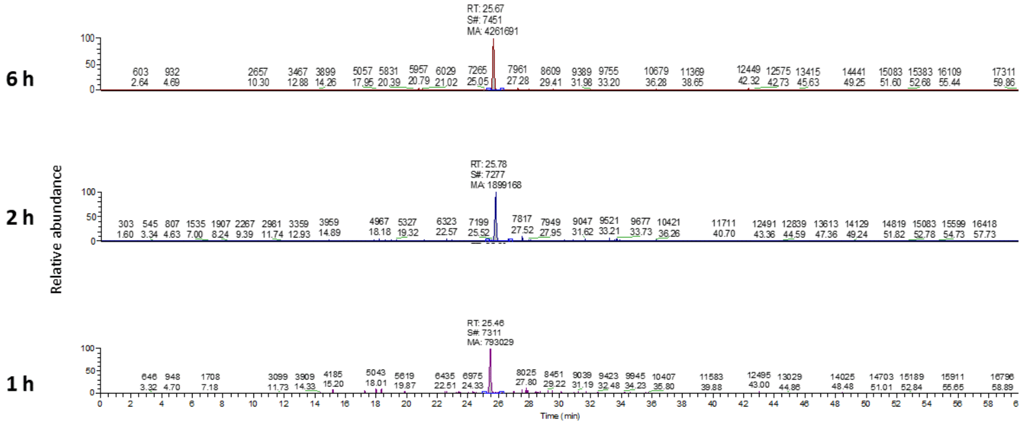

Figure S9. Mass spectrometry quantitative analysis of the Pr-CoA-propionylated peptides containing K524. The effective AUC (areas under the curves) was in gray.
